# Chemometrics, health risk assessment and probable sources of soluble total petroleum hydrocarbons in atmospheric rainwater, Rivers State, Nigeria

**DOI:** 10.1038/s41598-022-15677-7

**Published:** 2022-07-12

**Authors:** Daniel Omeodisemi Omokpariola, John Kanayochukwu Nduka, Henrietta Ijeoma Kelle, Nkoli MaryAnn Mgbemena, Emily Osa Iduseri

**Affiliations:** 1 Environmental Chemistry and Toxicology Unit, Pure and Industrial Chemistry Department, Nnamdi Azikiwe University, P.M.B. 5025, Awka, Nigeria; 2 Department of Chemistry, Faculty of Science, National Open University of Nigeria, Abuja, Nigeria; 3 Department of Chemistry, Michael Okpara University of Agriculture, Umudike, Abia State Nigeria; 4 Department of Environmental Sciences, Faculty of Science, National Open University of Nigeria, Abuja, Nigeria

**Keywords:** Environmental sciences, Chemistry

## Abstract

Total petroleum hydrocarbons (TPHs)—(aliphatic and aromatic) were analysed for in atmospheric rainwater between April–June; July–August; September–October depicting early, mid, late rain of 2019. Sampling at Rumuodomaya/Rumuodome and Ogale in Rivers State using basins fastened to a Table 2M above ground and 120 M from high features, Rainwater was analysed after treatment using Agilent GC-FID. Results show cumulative TPHs at R/R were 56.6551 mg/L, 39.5201 mg/L and 7.2283 mg/L, Ogale: 9.1217 mg/L, 59.4923 mg/L and 21.9825 mg/L. Aliphatic hydrocarbons: C5–C8 were < 1, low contamination, other carbon aggregates (C9–C16, C17–C35, and C36–C40) indicate high contamination. Chemometric assessment showed high contamination. TPHs aggregates at Rumuodomaya/Rumuodome were–C8–C11 (1.034 and 1.005) early rain, C18–C25 and C26–C33 has Carbon preference index of 1.287 and 1.630 (mid-rain), C26–C33 has CPI of 1.288 (late-rain), Ogale area, C26–C33 has CPI of 1.732 (early-rain), mid-rain C8–C11 (2.768) and C12–C17 (5.368). Pristane/phytane ratio indicated biogenic and pyrogenic sources. Average carbon length of TPHs for odd n-alkanes were C9–C11 (9.446) and C35–C39 (38.980), C9–C11(10.238), C35–C39 (36.510); C9–C11 (10.240) and C35–C39 (36.934). Average daily intake depicted possible health issues for children and adults as hazard index > 1 for aromatics.

## Introduction

Rivers State Nigeria has been a petroleum exploration and exploitation hotspot for over 50 years covering a land mass of about 10,575 square kilometres^1^. There is continuous wet and dry season through the year resulting from the impact of the Atlantic Ocean and Sahara Desert continental air masses. Annual rainfall ranges from 1900–2850 mm, temperature varies from 22.6–34.0 °C, relative humidity between 65–80%^1^. Different local and international oil companies situated onshore and offshore with downstream petroleum depots and filling stations surround major oil-producing communities and connect several pipelines and flow stations. These activities may likely result in the release of environmental contaminants such as polynuclear aromatic hydrocarbons (PHAs), aliphatic hydrocarbons, heavy metals, organometallic compounds, aerosols, ashes, particulates, H_2_S, CO_X_, NO_X_, SO_X_, soot, smoke, etc. into the atmosphere as activities of petroleum companies are intense in Nigeria’s crude oil and gas-bearing region reputed to house several billions of crude oil and trillions of cubic feet of natural gas^2^. Out of several environmental contaminants that may be released, total petroleum hydrocarbons (TPHs) stand out and are chemical compounds produced by phytogenic (plants, animal and microbes), petrogenic (crude oil, gas, and coal), and pyrogenic (natural and anthropogenic) combustion of carbonaceous component^3^.

They are made from carbon and hydrogen compounds that range from C_5_–C_40_ with aromatic (cyclic ring) such as polycyclic aromatic hydrocarbons, BTEX (benzene, toluene, ethylbenzene, and xylene), asphaltenes, prophrins, NSO (nitrogen, sulphur, oxygen) compounds, etc. and aliphatic saturates (straight-chain, branched-chain and cyclic alkanes) such as paraffins, isoprenoids, naphthenes, etc.^4^. Suitable environmental conditions can activate the formation of toxic polychlorinated-n-alkanes of formula CnH_2_n + _2-2_X_2_ (X is chlorine of halogen group)^5^, C10–C13 carbon chain has the most probable environmental emission^6^, as a result of photolysis, hydrolysis, oxidation, and biodegradation (degradation processes) transform chemical compounds upon entry into its environmental recipient, They are released across environmental matrices (lithosphere, hydrosphere, and atmosphere) by human activities such as petroleum exploration and drilling process, industrial effluents, accidental spills, automobile releases, petroleum transport and depot storage, which causes tremendous contamination over the cumulative period^7–9^. Pyrogenic, petrogenic, and phytogenic emission into the atmosphere leads to high deposition of particulates on different surfaces.

Rainfall washes these particulates and gases out from the atmosphere. Several assessments have shown that TPH influences inorganic parameters of water such as pH, temperature, dissolved oxygen, total solids, and turbidity already reported^7,10^. A higher amount of these petroleum chemicals reacts physiochemically and biologically across environmental matrices leading to the production of persistent organic pollutants (POPs), micro particulates, and sediments triggering a high risk of bioaccumulation in humans that may result in mental, genetic, immune, endocrine-disruption, respiratory, teratogenic and carcinogenic effects^11,12^. Previous studies have focused on TPHs in soil and sediment, aquatic species, and marine sources^13–15^, while polyaromatic hydrocarbon (PAHs) in soils and surface water of the region has received voluminous literature^16,17^ and consistently proved the negative impact to flora and faunas of the study area by organic emissions from various activities previously enumerated above, however, we are not aware of any literature evidence of TPH via atmospheric rainwater deposition in Nigeria, this forms the basis of our research. The reason for this work is to (i) to estimate the TPHs in atmospheric rainwater in crude oil and gas region of Nigeria (ii) source identification (iii) evaluation of carbon preference index and average carbon length and (iv) non-carcinogenic risk assessment of TPH.

## Materials and methods

### Sample collection and preparation

Rainwater samples were collected from four quadrants (North, West, East, South) ambient (open-air) in Ogale (O/E), Eleme local government area (4.786414º N; 7.139266º E) and Rumuodomaya/Rumuodome (R/R), Obio-Akpor local government area (4.856923º N; 7.014726º E) in Rivers State, Nigeria between the months of April–May (early rain), June–August (mid rain) and September–October (late rain) in 2019, hence sample duration was April–October (7 months) by using pre-cleaned amber glass bottles. The four (4) samples per sampling regimen were thereafter homogenised together using equal mass (500 ml) to get a final sample which was labelled, four (4) samples per period and per location, given a total of twenty four (24) at different locations of Rumuodomaya/ Rumuodome (R/R) and Ogale (O/E) in Rivers State, Nigeria using basins fastened to a Table 2M above ground and 120 M from high rise features, atmospheric rainwater after collection, was filtered into amber glass bottles before analysis. The samples were labelled, packaged in a black cellophane bag, which was taken to the laboratory for analysis.

### Extraction of petroleum hydrocarbon from rainwater samples

The extraction of petroleum hydrocarbons from rainwater samples was carried out using the United States Environmental Protection Agency (US EPA) methodology 8015D. Precisely 200 mL of rainwater samples was measured out into a pre-cleaned separating funnel and mixed with 50 mL of dichloromethane. The mixture in the separating funnel was mechanically shaken for five minutes and allowed to separate into organic and aqueous phases; thereafter the organic phase was collected into a beaker and repeated thrice with 10 mL dichloromethane (DCM) continuously. The corresponding organic fractions were mixed into a beaker and subsequently concentrated using a rotary concentrator^4,18^.

### Gas Chromatography analysis

The petroleum hydrocarbons were quantified using Agilent GC-FID 7820A (gas chromatography equipped with a flame ionization detector). The GC-FID column infused with HP-5 fused silica capillary having dimension (30 m × 0.32 mm × 0.25 μm film thickness) with helium gas as carrier gas at 1.75 mL/min flow rate. The detection limit of the instrument (Agilent GC-FID) is ≥ 0.0001 across the petroleum hydrocarbon mixture. Appropriate calibration was conducted using hydrocarbon standard mixture with integrated limit from C_8_H_18_ to C_40_H_82_ with seven calibration-level prepared (0.01 mg/l, 0.5 mg/l, 2.5 mg/l, 10 mg/l, 50 mg/l, 100 mg/l, 150 mg/l) and injected into the gas chromatogram to obtain calibration curve of 0.99978 correspondingly. The concentrated sample extract was injected in splitless mode using DCM as standard to remove background interference to chromatogram area, as the temperature followed a stepwise process: initial temperature (50 °C) holds for 2 min, steady increase from 10 to 100 °C, holds for 2 min then ramp at 5 °C to 250 °C and hold for 3 min, the final ramp from 5 to 320 °C and hold for 10 min^18^. The final chromatogram results were analyzed with the Agilent software (chemstation).

The detection limit (≥ 0.0001) of an instrument is dependent on agilent GC-FID manufacturing processes in relation to calibration curve from total carbon fraction mixture (C6-C40) which has been stated already in that it is used as a reference value for detecting TPHs in a sample (rainwater). The instrument has a dictionary called chemstation (an agilent dictionary) which tells the analyst the amount seen from the FID detector after GC separation. 

### Chemometric assessment

#### Contamination factor and pollution load index

The contamination factor, CF is calculated to reveal the extent of aggregate TPHs contamination in rainwater, while pollution load index entails the comparative means to assess the cumulative level of TPHs for both aliphatic and aromatic forms using reference values

1 $${\text{CF}} = \frac{{{\text{C}}_{{\text{s}}} }}{{{\text{C}}_{{\text{r}}} }}$$

where, Cs = concentration of aggregate TPHs (mg/L), Cr = reference standards using TPHCWG^19^.

Mathematically, it is expressed as CF ≤ lower contamination, 1 < moderate, 3 < Considerable, 6 ≥ CF, high contamination^20^. The reference standards are shown in Table 1.

**Table 1:** Reference standards for petroleum hydrocarbons.

Aliphatic	Reference values (mg/L)
C5–C8	3
C9–C16	0.065
C17–C35	1
C36–C40	10

Aromatic	Reference values (mg/L)
C5–C8	0.13
C8–C16	0.025
C17–C35	0.02
C36–C40	0.2

TPHCWG, 2019.

The Pollution Load Index (PLI) is given as:

2 $$\mathrm{PLI}=({\mathrm{CF}}_{1}\times {\mathrm{CF}}_{2}\times \cdots \times {\mathrm{CF}}_{\mathrm{n}}{)}^{1/\mathrm{n}}$$

where CF_1_ = contamination factor of each TPH aggregate assessed, n = number of components assessed.

#### Principal component assessment

Principal component assessment (PCA) is a statistical tool used to identify various matrices to derive salient information about components. PCA is a data reduction technique used to find the linear combinations of variables (TPHs) leading to the formation of factors^21^, gives un-rotated and rotated varimax, as rotated varimax is commonly utilized because it facilitates the best interpretation from sets of variables. The rotation is simply a process that allows a new axis to be chosen while maintaining individual components^22^. They are graded as strong (> 0.75), moderate (0.75–0.50), and weak (0.50–0.30) as done by Liu et al.^23^. PCA was determined using Microsoft Excel with XL-STAT add-ins, 2019.

### Petroleum hydrocarbon identification using molecular makers

#### Carbon preference index

Carbon preference index (CPI) is the total of odd n-alkanes divided by the sum total of even n-alkanes for a range of C_8_–C_40_ to estimate the relative source identification of either natural or artificial contribution to petroleum hydrocarbon.

3 $$\mathrm{CPI }= (\mathrm{sum\,of\,odd\,n}-\mathrm{alkanes}) / (\mathrm{sum\,of\,even\,n}-\mathrm{alkanes})$$

CPI is used as a forensic indicator for petroleum source identification of TPHs. CPI values greater than 1 shows the natural contribution from biological phytoplankton plants while CPI less than 1 shows the anthropogenic (artificial) source from ubiquitous contribution^24,25^. TPHs were aggregated into five forms to conduct CPI evaluation: C_8_–C_11_, C_12_–C_17_, C_18_–C_25_, C_26_–C_33,_ and C_34_–C_40_.

#### Average carbon length (ACL)

Average carbon length is a guide used to evaluate odd n-alkanes dominance per molecule in environmental samples to form link to petrogenic plant source as a forensic tool to assess hydrocarbon contamination^26^. ACL was aggregated into five categories used in CPI assessment and calculated using Eq. (4) below

4 $$\mathrm{For }{\mathrm{C}}_{9}-{\mathrm{C}}_{11};\mathrm{ ACL}= \frac{ 9\left({\mathrm{nC}}_{9}\right)+11\left({\mathrm{nC}}_{11}\right)}{{\mathrm{C}}_{9}+{\mathrm{C}}_{11}}$$

The formula, as shown above, was conducted for other aggregates respectively. ACL values are usually constant in unpolluted rainwater samples but fluctuate as values deplete in polluted rainwater^27^. ACL was assessed for even n-alkanes in the environment to assess the anthropogenic impact in the environment.

#### Long-chain hydrocarbon/short-chain hydrocarbons (LHC/SHC)

Long-chain hydrocarbons are n-alkanes above n- alkanes at C_26_ usually from vascular plant-based anthropogenic sources, while short-chain hydrocarbons are n-alkanes below n-alkanes at C26 from phytoplankton or algae-based sources^28^.

5 $$\frac{{{\text{LHC}}}}{{{\text{SHC}}}} = \left( {\frac{{\sum > {\text{nC}}_{{26}} }}{{\sum < {\text{nC}}_{{26}} }}} \right)$$

They are assessed using odd and even n-alkanes. For odd n-alkanes, LHC/SHC between 0–1.00 shows phytoplankton, < 4.00 shows a mixture of phytoplankton and terrestrial plants, > 4.00 indicates terrestrial plant inputs. For even n-alkanes, LHC/SHC between 0–1.00 shows inputs from anaerobic microbial biogenic sources, < 4.00 shows a mixture between anaerobic and anthropogenic petroleum, releases > 4.00 shows that anthropogenic petroleum releases.

#### Low molecular weight/ high molecular weight (L/H)

It is the ratio of low molecular weight n-alkanes below C20, while above C20 gives the high molecular weight used to determine the n-alkanes. L/H values close to unity (1) show natural input from marine and terrestrial biological sources, while above one (1) indicates inputs from petroleum sources^29^.

#### C_31_/C_19_ ratio

It is used as source identification and differentiation for TPHs in rainwater. C31 is assumed to be from terrestrial biogenic hydrocarbons, while C19 proposes marine inputs. The ratio below 0.4 reveals marine sources, while above 0.4 is from terrestrial biogenic or non-marine sources^30^.

#### Pristane/phytane ratio

It is the ratio of the abundance of pristane to phytane for redox conditions in the aquatic environment, based on the assumption that pristane is from oxygenated (aerobic) source degradation of planktons, while phytane is from reduction (anaerobic) source degradation of planktons by microbes. As such, Pristane/phytane ratio below unity (1) indicates oxic condition (pyrogenic source), while greater than 1 indicates anoxic (biogenic source)^31^.

### Human health risk assessment

Human health risk assessment (HHRA) is a tool used to estimate if common and potential contaminants released into the environment will adversely affect the health of humans over a long period from diverse exposure paths (inhalation, injection, dermal). According to Bharadwaj and Machibroda^32^, HHRA may not prove that diseases are connected to an exposure pathway or a particular chemical agent as humans are exposed to numerous chemical agents. According to USEPA^33^, it is based on characterization, risk assessment, hazard identification, receptor characterization, exposure assessment, and risk characterization.

In this study, non-carcinogenic HHRA were evaluated for adults and children for TPHs exposure via dermal and ingestion using USEPA models as shown in Eq. 6 and 7^8,33^.

6 $${\mathrm{ADI}}_{\mathrm{dermal}}= \frac{{\mathrm{C}}_{\mathrm{w}}\times \mathrm{SAF}\times \mathrm{SA}\times \mathrm{DAF}\times \mathrm{ED}}{\mathrm{BW}\times \mathrm{AT}\times \mathrm{GIABS}\times \mathrm{THQ}}$$

7 $${\mathrm{ADI}}_{\mathrm{ingestion}}= \frac{{\mathrm{C}}_{\mathrm{w}}\times {\mathrm{IR}}_{\mathrm{w}}\times \mathrm{ED}}{\mathrm{BW}\times \mathrm{AT}\times \mathrm{THQ}}$$

$$\mathrm{Where}:\mathrm{ ADI is the Average daily intake }\left({\mathrm{mgkg}}^{-1}{\mathrm{day}}^{-1}\right).$$

$${\mathrm{C}}_{\mathrm{w}}\mathrm{ is the concentration of petroleum hydrocarbons in water }(\mathrm{mg}/\mathrm{L}).$$

$$\mathrm{SAF is skin adherence factor};\left(0.12{\mathrm{mgcm}}^{-2}\mathrm{ for adults and }0.2{\mathrm{mgcm}}^{-2}\mathrm{ children}\right).$$

$$\mathrm{SA is exposed skin area}: \left(2373{\mathrm{cm}}^{2}/\mathrm{day for adults and }3527{\mathrm{cm}}^{2}/\mathrm{day for children}\right)$$

$$\mathrm{DAF is dermal absorption factor }\left(\mathrm{unitless}\right);\left(0.1\mathrm{ for adults and children}\right)$$

$$\mathrm{ED is exposure duration};\left(25\mathrm{ years for adults and }6\mathrm{ years for children}\right).$$

$$\mathrm{BW is body weight};\left(80\mathrm{kg for adults and }15\mathrm{kg for children}\right).$$

$$\mathrm{AT is the average time for non}-\mathrm{carcinogen}=\mathrm{ ED}\times 365\mathrm{ days}$$

$$\mathrm{GIABS is a gastrointestinal absorption factor }\left(\mathrm{unitless}\right):\left(1.0\mathrm{ for adults and children}\right).$$

$$\mathrm{THQ is target noncancer hazard quotient}: \left(1.0\mathrm{ for adults and children}\right).$$

$${\mathrm{IR}}_{\mathrm{w}}\mathrm{ is ingestion rate}; \left(2{\mathrm{L day}}^{-1}\mathrm{ for adults and }1\mathrm{L }{\mathrm{day}}^{-1}\mathrm{ for children}\right).$$

The chronic (average) daily intake obtained from Eq. (6) and Eq. (7) were used to obtain the hazard index for non-carcinogenic TPHs as shown below:

8 $${\text{HI}} = {\text{HQ}}_{{{\text{dermal}}}} + {\text{~HQ}}_{{{\text{ingestion}}}} = \left[ {\left( {\frac{{{\text{ADI}}_{{{\text{dermal}}}} }}{{{\text{RfD}}}}} \right)} \right] + \left[ {\left( {\frac{{{\text{ADI}}_{{{\text{ingestion}}}} }}{{{\text{RfD}}}}} \right)} \right]$$

where Hazard Index (HI) is the total of Hazard quotient (HQ) from dermal and ingestion, as the acceptable limit is 1.0^34^.

Hazard quotient is the probability that an adverse health effect is imminent (unitless).

The reference dose (RfD) is shown in Table 2.

**Table 2:** Reference dose of total petroleum hydrocarbons (TPHs).

Aliphatic	Dermal	Source	Ingestion	Source
Low carbon range (C5–C8)	5	TPHCWG, 2019	0.04	CEPA, 2009
Medium carbon range (C9–C18)	0.1	TPHCWG, 2019	0.01	USEPA, 2016
High carbon range (C19–C32)	2	TPHCWG, 2019	3	USEPA, 2016
High carbon range (C33–C40)	20	TPHCWG, 2019	30	PPRTV
Aromatic	Dermal	Source	Ingestion	Source
Low carbon range (C6–C16)	0.2	TPHCWG, 2019	0.004	USEPA, 2016
Medium carbon range (C17–C32)	0.04	TPHCWG, 2019	0.04	USEPA, 2016
High carbon range (C33–C40)	0.03	TPHCWG, 2019	0.4	HEAST

*TPHCWG* Total Petroleum Hydrocarbon Criteria Working Group, *PPRTV* Provisional Peer-Reviewed Toxicity Value, *HEAST* Health Effect Assessment Summary Table.

### Consent for Publication

All the authors agreed to submit the manuscript to Scientific Report for consideration and possible publication.

## Results

Table 3 mean concentration of total petroleum hydrocarbons (C_8_–C_40_) assessed in Rumuodomaya/Rumuodome, Obio-Akpor LGA (R/R), and Ogale, Eleme LGA (O/E). The total of early, mid, and late rain of R/R are 56.6551 mg/L, 39.5201 mg/L, and 7.2283 mg/L, while O/E were 9.1217 mg/L, 59.4923 mg/L, and 21.9825 mg/L. For carbon chain length of C_8_–C_40_ at R/R, lower carbon length has highest value of C_9_ = 0.0948(early rain), C_13_ (2.2727) mid rain, C_10_ (0.3963) late rain. Longer chain highest values were C39 (4.3094), C_40_ (51.3652) all for early rain, that of mid and late rain at C40 (6.5533 and 1.6351). At Ogale area, lower carbon chain highest values were as C_9_ (0.7001) early rain, C_9_ (0.7284, mid-rain), C_9_ (0.0827, late rain) but longer chain highest values are C_40_ (1.8601, 22.237) for early and late rain but highest for late rain is C_34_ (4.7606).

**Table 3:** Mean concentration of TPHs determined in rainwater samples.

TPHS	Rumuodomaya/Rumuodome, Obio-Akpor local government area (LGA)			Ogale, Eleme local government area		
	Early rain	Mid rain	Late rain	Early rain	Mid rain	Late rain
C8	0.0419	0.0683	0.1138	0.6713	0.0342	0.0211
C9	0.0948	0.9477	0.1848	0.7001	0.7284	0.0827
C10	0.0498	1.4817	0.3963	0.2850	0.2290	0.0344
C11	0.0272	1.5403	0.3017	0.3225	0.1403	0.0183
C12	0.0294	1.3199	0.3533	0.3214	0.2550	0.0179
C13	0.0121	2.2727	0.2963	0.1228	0.1380	0.0159
C14	0.0245	1.6737	0.1360	0.2343	0.0664	0.0506
C15	0.0098	0.8761	0.3332	0.3279	0.0571	0.0365
C16	0.0161	1.3738	0.2074	0.1304	0.0291	0.1412
C17	0.0090	0.4859	0.0463	0.1998	1.6863	0.2815
Pristane	0.0120	0.8945	0.2664	0.1118	2.4970	0.0775
C18	0.0208	1.0053	0.1172	0.4235	2.5371	0.4606
Phytane	0.0086	1.6272	0.1383	0.2979	1.8832	0.2612
C19	0.0122	1.3125	0.1710	0.0500	1.3617	0.5663
C20	0.0233	0.7409	0.2457	0.0513	2.2847	0.5956
C21	0.0046	0.9557	0.1274	0.1906	1.6645	0.1990
C22	0.0193	1.3712	0.2059	0.2061	1.0623	0.2579
C23	0.0280	1.2082	0.1362	0.1010	2.8227	0.6480
C24	0.0100	1.1233	0.2084	0.2274	1.9995	0.5236
C25	0.0290	1.9830	0.1881	0.3157	0.8231	0.7794
C26	0.0222	1.1604	0.1231	0.1463	2.2010	0.9508
C27	0.0050	1.7674	0.1348	0.2968	0.9965	1.1551
C28	0.0198	0.4857	0.1242	0.0924	1.5103	0.9357
C29	0.0122	0.6331	0.1461	0.2119	0.2411	0.7200
C30	0.0061	0.4414	0.0757	0.0746	1.1923	0.4986
C31	0.0093	1.2852	0.1376	0.0735	1.0964	2.4430
C32	0.0093	0.3896	0.0958	0.0866	1.4583	1.0476
C33	0.0131	0.3519	0.1211	0.1106	1.1497	1.6139
C34	0.0047	0.2803	0.0142	0.1100	0.7749	0.5486
C35	0.0187	0.5108	0.1129	0.2260	0.9056	0.6931
C36	0.0095	0.7341	0.0997	0.1129	0.6614	4.7606
C37	0.0057	0.113	0.0950	0.1438	0.7364	0.0050
C38	0.3627	0.2630	0.0367	0.1541	0.4592	0.0113
C39	4.3094	0.2877	0.1026	0.1367	1.2048	0.0180
C40	51.3652	6.5533	1.6351	1.8601	22.2371	1.5107
TOTAL	56.6551	39.5201	7.2283	9.1271	59.4323	21.9825

N = 4.

Table 4 shows contamination factor (CF) conducted with pollution load index (PLI). The cumulative CF were 9.953, 195.016, and 36.751 for R/R, while O/E were 41.265, 55.5908, and 21.6713 for aliphatic petroleum hydrocarbons. Aromatic petroleum hydrocarbons at R/R were 304.96, 1374.307, and 225.666 for early, mid, and late rain; while O/E were 274.683, 1580.89, and 793.505 accordingly. Considering aliphatic hydrocarbon shows that the more volatile C_5_–C_8_ were not significantly present in the sample regimens. Highest values for R/R study area were C_9_–C_16_ (4.0569, 176.706, 33.9846) for early, mid and late rain respectively. C36–C40 had 5.6053 for early rain. For Ogale study area, highest values were C_9_–C_16_ and C_17_–C_35_ (37.6062, 25.2815, 6.1154 and 3.1941, 27.7680, 14.9183) depicting early, mid and late rain values. When considering aromatic hydrocarbon for R/R area, C_9_–C_16_ (459.436 and 88.360) and C_17_–C_37_ (874.590 and 126.585) for mid and late rain. C_36_–C_40_ (280.2600 and 39.7560) for early and mid-rain. At Ogale study area C_17_–C_35_ (159.7050, 1388.40, 745.915) for the three regimens of sampling. The mean contamination factor and pollution load index of aliphatic hydrocarbon were CF (2.4882, 48.7540, 9.1875) and PLI (0.5444, 2.7350, 0.8977) for R/R area. At Ogale area; CF (10.3160, 13.8977, 5.4178) PLI (1.5949, 2.1211, 0.7976) all for early, mid and late rain. For aromatic hydrocarbon at both R/R and Ogale study area CF (76.241, 343.577, 56.4165), PLI (10.714, 53.824, 17.6205) and CF (68.6707, 395.222, 198.3763), PLI (31.3884, 41.7426, 156,957).

**Table 4:** Contamination Factor and Pollution Load Index of Petroleum hydrocarbons aggregates.

		Rumuodomaya/Rumuodome, Obio-Akpor LGA			Ogale, Eleme LGA		
		Early rain	Mid rain	Late rain	Early rain	Mid rain	Late rain
Aliphatic hydrocarbons	C5–C8	0.0140	0.0228	0.0379	0.2238	0.0114	0.0070
	C9–C16	4.0569	176.7060	33.9846	37.6062	25.2815	6.1154
	C17–C35	0.2766	17.4918	2.5317	3.1941	27.7680	14.9183
	C36–C40	5.6053	0.7951	0.1969	0.2408	2.5299	0.6306
	Mean CF	2.4882	48.7540	9.1878	10.3160	13.8977	5.4178
	Σ CF	9.9527	195.0160	36.7510	41.2648	55.5908	21.6713
	PLI	0.5444	2.7350	0.8954	1.5949	2.1211	0.7976
Aromatic hydrocarbons	C5–C8	0.3223	0.5254	0.8754	5.1638	0.2630	0.1623
	C9–C16	10.5480	459.4360	88.3600	97.7760	65.7320	15.9000
	C17–C35	13.8300	874.5900	126.5850	159.7050	1388.4000	745.9100
	C36–C40	280.2600	39.7560	9.8455	12.0380	126.4940	31.5280
	Mean CF	76.2410	343.5770	56.4165	68.6707	395.2220	198.3763
	Σ CF	304.9600	1374.3070	225.6660	274.6830	1580.8900	793.5053
	PLI	10.7140	53.8240	17.6205	31.3884	41.74260	15.6957

Table 5 shows principal component analysis conducted for TPHs mean concentration across the two locations. Factor analysis gave two factors for rotated varimax. R/R had cumulative variance at 29.50%, the rotated varimax for C_8_–C_40_ ranged from − 0.995 to 0.998. O/E had cumulative variance at 77.89% with rotated varimax for C_8_–C_40_ ranged from − 0.667 to 0.995. The statistical analysis showed presence of positive and negative correlation for both R/R and O/E respectively. As such, one can see that in R/R, Factor 1 had predominantly positive correlation (0.103–0.821), which implies that carbon substrates (C9–C37) were from similar contamination source and biochemical interaction and vice versa, Factor 2 (− 0.571 to − 0.995) for negative correlation due to dissimilar sources. For O/E, there was presence of negative correlation for both Factor 1 and 2 via C8–C40, which implies a combination of similar and dissimilar contamination in tandem with biochemical interaction.

**Table 5:** Principal component analysis of mean concentration of TPHs in rainwater.

	Rumuodomaya/ Rumuodome, Obio-Akpor Local Government Area (LGA)		Ogale, eleme local government area (LGA)	
	Rotated varimax		Rotated varimax	
	1	2	1	2
C8	− 0.667	− 0.745	0.4673	0.884
C9	0.781	− 0.624	− 0.55	0.835
C10	0.688	− 0.726	− 0.323	0.946
C11	0.732	− 0.681	0.0939	0.996
C12	0.681	− 0.732	− 0.328	0.945
C13	0.769	− 0.639	− 0.611	0.791
C14	0.803	− 0.596	0.4134	0.911
C15	0.577	− 0.817	0.4264	0.905
C16	0.759	− 0.651	0.9977	− 0.068
C17	0.797	− 0.604	− 0.998	− 0.069
Pristane	0.651	− 0.759	− 1.000	− 0.007
C18	0.786	− 0.618	− 0.999	− 0.035
Phytane	0.796	− 0.605	− 1.000	8E−05
C19	0.771	− 0.637	− 0.913	− 0.409
C20	0.633	− 0.774	− 0.968	− 0.253
C21	0.767	− 0.642	− 1.000	− 0.025
C22	0.761	− 0.648	− 0.997	− 0.074
C23	0.789	− 0.614	− 0.978	− 0.209
C24	0.735	− 0.679	− 0.984	− 0.175
C25	0.795	− 0.606	− 0.549	− 0.836
C26	0.791	− 0.612	− 0.914	− 0.407
C27	0.800	− 0.600	− 0.323	− 0.946
C28	0.702	− 0.712	− 0.795	− 0.607
C29	0.708	− 0.706	0.4725	− 0.881
C30	0.747	− 0.665	− 0.919	− 0.394
C31	0.784	− 0.62	0.098	− 0.995
C32	0.699	− 0.715	− 0.717	− 0.697
C33	0.625	− 0.78	− 0.196	− 0.981
C34	0.821	− 0.571	− 0.748	− 0.664
C35	0.725	− 0.688	− 0.727	− 0.686
C36	0.770	− 0.638	0.4215	− 0.907
C37	0.103	− 0.995	− 0.987	0.159
C38	0.716	0.698	− 0.956	0.293
C39	0.093	0.996	− 0.997	0.071
C40	0.144	0.99	− 1	− 0.005
Eigenvalue	17.60	17.40	20.799	14.201
Variance (%)	8.717	20.782	33.681	41.204
Cumulative Variance (%)	8.717	29.499	33.681	77.885

Table 6 shows carbon preference index (CPI) conducted for five TPH aggregates (C_8_–C_11_, C_12_–C_17_, C_18_–C_25_, C_26_–C_33_, C_34_–C_40_). R/R ranged from 0.083 to 1.630 for early, mid and late rain regiments, while O/E ranged from 0.105 to 5.368. The CPI of TPHs aggregate highest values shows that C_8_–C_11_ and C_18_–C_25_ (1.034 and 1.005) early rain, C_18_–C_25_ and C_26_–C_33_ (1.287 and 1.630) mid-rain, C_26_–C_33_ (1.288) late rain for Rumuodomaya/Rumuodome (R/F) while at Ogale, the highest values were C_26_–C_33_ (1.732) early rain, C_8_–C_11_ and C_12_–C_17_ (2.768 and 5.368) mid-rain but at late rain, the highest values were more depicted as C_8_–C_11_, C_12_–C_17_, C_18_–C_25_ and C_26_–C_33_ (1.490, 1.592, 1.193 and 1.728). In all, about 40% of carbon chain aggregates were above standard carbon preference index of one (1) at both study locations and sampling regimen.

**Table 6:** Carbon preference index (CPI) of TPHs aggregates.

	Rumuodomaya/rumuodome, Obio-Akpor LGA			Ogale, Eleme LGA		
	Early rain	Mid rain	Late rain	Early rain	Mid rain	Late rain
C8–C11	1.034	0.611	0.362	0.732	2.768	1.490
C12–C17	0.441	0.832	0.970	0.948	5.368	1.592
C18–C25	1.005	1.287	0.801	0.724	0.846	1.193
C26–C33	0.69	1.630	1.288	1.732	0.548	1.728
C34–C40	0.084	0.116	0.174	0.226	0.118	0.105
CPI standard	1	1	1	1	1	1

Table 7: The average carbon length (ACL) conducted for five TPH aggregates (C_8_–C_11_, C_12_–C_17_, C_18_–C_25_, C_26_–C_33_, C_34_–C_40_). The ACL values for R/R ranged from 8.596 to 39.66 for odd and even n-alkanes derivatives, while O/E ranged from 9.086 to 39.985 respectively. Odd n-alkanes, C_19_–C_25_, C_27_–C_33_ and C_35_–C_39_ had highest average carbon length of 23.00, 30.540, 38.980 (early rain), 22.415, 29.110, 36.510 (mid-rain), 22.097, 29.908, 36.934 (late rain) for R/R area. At Ogale, considering the same carbon chain range, 23.076, 28.994, 36.647 (early rain), 21.931, 30.377, 37.210 (mid-rain), 22.496, 30.522, 35.115 (late rain). For even n-alkanes, following the same carbon chain length (C_19_–C_25_, C_27_–C_33_ and C_35_–C_39_) 20.504, 28.087, 39.985 (early rain); 21.232, 28.508, 39.688 (mid-rain); 21.30, 28.689, 39.688 (late rain) of R/R study area. Ogale study area is depicted as 20.508, 28.508, 39.365 (early rain); 20.640, 28.600, 39.660 (mid-rain); 20.919, 28.957, 36.727 (late rain), same carbon chain length C_18_–C_24_˂C_26_–C_32_˂C_34_–C_40_ as expected due to the volatile nature of lower fractions.

**Table 7:** Average carbon length (ACL) for TPHs aggregates.

	Rumuodomaya/ Rumuodome, Obio-Akpor LGA			Ogale, Eleme LGA		
	Early rain	Mid rain	Late rain	Early rain	Mid rain	Late rain
**Odd n-alkanes**						
C9–C11	9.446	10.238	10.240	9.631	9.323	9.362
C13–C17	14.799	14.017	14.260	15.237	16.646	16.591
C19–C25	23.000	22.415	22.097	23.076	21.931	22.496
C27–C33	30.540	29.110	29.908	28.994	30.377	30.522
C35–C39	38.980	36.510	36.934	36.647	37.210	35.115
**Even n-alkanes**						
C8–C10	9.086	9.912	9.553	8.596	9.740	9.240
C12–C16	13.620	14.025	13.581	13.440	12.711	15.176
C18–C24	20.504	21.232	21.301	20.523	20.640	20.919
C26–C32	28.087	28.049	28.689	28.508	28.600	28.957
C34–C40	39.985	39.340	39.688	39.365	39.660	36.727

Table 8: Long chain hydrocarbons/short chain hydrocarbons (LHC/SHC) for C_8_–C_40_. The odd n-alkanes values R/R for early, mid and late rains were 22.268, 0.722, and 0.65, O/E were 0.752, 0.832, and 4.02. For even n-alkanes values, R/R were 220.33, 1.015, and 1.111, while O/E were 1.034, 3.589, and 4.881. The ratio of pristane/phytane values were higher at early rain (1.3953), late rain (1.9262) for R/R study area and mid -rain (1.3259) for Ogale area. LHC/SHC (odd n-alkanes) ratio were of significant value of 22.268 (early rain) and 4.019 (late rain) for R/R and Ogale respectively, while the LHC/SHC (even n-alkane) ratio were all significant at all sampling regimen at both study location but mostly pronounced at the early rain (220.33) of R/R area.

**Table 8:** Petroleum hydrocarbons source diagnostic ratios.

	Rumuodomaya/Rumuodome, Obio-Akpor LGA			Ogale, Eleme LGA		
	Early rain	Mid rain	Late rain	Early rain	Mid rain	Late rain
TPHs	56.655	39.520	7.228	9.127	59.432	21.983
C_31_/C_19_	0.762	0.979	0.805	1.470	0.805	4.314
L/H	0.007	0.690	0.740	0.787	0.211	0.120
Pristane/Phytane	1.395	0.550	1.926	0.375	1.326	0.297
ACL (odd n-alkanes)	37.705	21.148	21.246	20.845	25.234	28.248
ACL (even n-alkanes)	39.854	26.591	27.164	26.129	33.565	31.733
LHC/SHC (odd n-alkanes)	22.268	0.722	0.650	0.752	0.832	4.019
LHC/SHC (even n-alkanes)	220.330	1.015	1.111	1.034	3.589	4.881

Table 9 shows average daily intake (ADI) of aliphatic and aromatic petroleum hydrocarbons (PHs) for adults across early rain, mid rain and late rain respectively. For aliphatic PHs, R/R ADI (ingestion) ranged from 4.20E−05 mg/kg/day to 5.62E−02 mg/kg/day, ADI (dermal) ranged from 4.08E−05 mg/kg/day to 5.47E−02 mg/kg/day. O/E ADI (ingestion) varies from 2.11E−05 mg/kg/day to 2.82E−02 mg/kg/day; ADI (dermal) varies from 2.06E−05 mg/kg/day to 2.74E−02 mg/kg/day. For aromatic PHs, R/R ADI (ingestion) ranged from 2.40E−04 mg/kg/day to 5.62E−02 mg/kg/day; ADI (dermal) varies from 2.34E−04 mg/kg/day to 5.47E−02 mg/kg/day. O/E ADI (ingestion) varies from 4.19E−04 mg/kg/day to 2.82E−02 mg/kg/day, ADI (dermal) ranged from 4.088E−04 mg/kg/day to 2.74E−02 mg/kg/day. The aliphatic petroleum hydrocarbon exposure through dermal contact were in critical values 1 × 10^–3^–1 × 10^–1^ (C_8_–C_40_) for early, mid and late rain while ingestion exposure ranged from 1 × 10^–6^–1 × 10^–4^ for the same sampling regimen. Aromatic hydrocarbon for the R/R area through skin exposure ranged from 1 × 10^–3^–10^–1^ but via ingestion is 1 × 10^–5^–1 × 10^–1^ but when considering Ogale study area, aliphatic hydrocarbon exposure through dermal ranged as 1 × 10^–4^–1 × 10^–1^ while ingestion is 1 × 10^–6^–1 × 10^–3^ across all carbon chain length for all sampling regimes but aromatic hydrocarbons via dermal contact ranged as 1 × 10^–3^–1 × 10^–1^ (R/R) while ingestion is 1 × 10^–5^–1 × 10^–3^.

**Table 9:** Average daily intake of TPHs exposure in rainwater for adults.

Location	TPHs group	TPHs derivatives	Exposure medium	Early rain	Mid rain	Late rain
Rumuodomaya/ Rumuodome, Obio-Akpor LGA	Aliphatic PHs	C6–C8	Ingestion	4.20E−05	6.84E−05	1.14E−04
			Dermal	4.09E−05	6.66E−05	1.11E−04
		C9–C18	Ingestion	2.94E−04	1.30E−02	2.38E−03
			Dermal	2.86E−04	1.27E−02	2.31E−03
		C19–C32	Ingestion	2.11E−04	1.49E−02	2.12E−03
			Dermal	2.05E−04	1.45E−02	2.07E−03
		C33–C40	Ingestion	5.62E−02	9.11E−03	2.22E−03
			Dermal	5.47E−02	8.87E−03	2.16E−03
	Aromatic PHs	C6–C16	Ingestion	3.06E−04	1.18E−02	2.33E−03
			Dermal	2.98E−04	1.13E−02	2.27E−03
		C17–C32	Ingestion	2.40E−04	7.96E−03	1.97E−03
			Dermal	2.34E−04	7.75E−03	1.92E−03
		C33–C40	Ingestion	5.62E−02	9.11E−03	2.22E−03
			Dermal	5.47E−02	8.87E−03	2.16E−03
Ogale, Eleme LGA	Aliphatic PHs	C6–C8	Ingestion	6.72E−04	3.42E−05	2.11E−05
			Dermal	6.55E−04	3.33E−05	2.06E−05
		C9–C18	Ingestion	3.07E−03	5.88E−03	1.14E−03
			Dermal	2.99E−03	5.72E−03	1.11E−03
		C19–C32	Ingestion	2.13E−03	2.07E−02	1.13E−02
			Dermal	2.07E−03	2.02E−02	1.10E−02
		C33–C40	Ingestion	2.86E−03	2.82E−02	9.18E−03
			Dermal	2.78E−03	2.74E−02	8.93E−03
	Aromatic PHs	C6–C16	Ingestion	3.12E−03	1.68E−03	4.19E−04
			Dermal	3.04E−03	1.64E−03	4.08E−04
		C17–C32	Ingestion	2.75E−03	2.50E−02	1.21E−02
			Dermal	2.68E−03	2.04E−02	1.18E−02
		C33–C40	Ingestion	2.86E−03	2.82E−02	9.18E−03
			Dermal	2.78E−03	2.74E−02	8.93E−03

Table 10 shows the average daily intake (ADI) of aliphatic and aromatic petroleum hydrocarbons (PHs) for children across three rain sampling regiments (early rain, mid rain and late rain). The minimum and maximum aliphatic PHs evaluated in R/R and O/E are ADI (ingestion): 3.85E−06 mg/kg/day and 1.02E−02 mg/kg/day, while for ADI (dermal): 2.72E−04 mg/kg/day and 7.23E−01 mg/kg/day. For aromatic PHs, the minimum and maximum ranges are ADI (ingestion): 4.39E−05 mg/kg/day and 1.02E−02 mg/kg/day, while ADI (dermal): 3.09E−03 mg/kg/day and 7.23E−01 mg/kg/day. Critical values of average daily intake of TPHs at R/R for aliphatic were C_33_–C_40_ (7.23E−01 and 1.17E−01) via dermal exposure for early and mid-rain. The same values were obtained for aromatic PHs through dermal contact for C_33_–C_40_ for early and mid-rain. At Ogale, critical values of aliphatic PHs were at C_19_–C_32_ (2.67E−01 and 1.46E−01) via dermal route for mid and late rain, C_33_–C_40_ (3.63E−01 and 1.18E−01) for the dermal contact with mid and late rain. Considering aromatic hydrocarbon critical values were C_17_–C_32_ (3.21E−01 and 1.55E−01) via dermal route to mid and late rain, also at C_33_–C_40_ (3.62E−01 and 1.18E−01).

**Table 10:** Average daily intake of TPHs exposure in rainwater for children.

Location	TPHs group	TPHs derivatives	Exposure medium	Early rain	Mid rain	Late rain
Rumuodomaya/ Rumuodome, Obio-Akpor LGA	Aliphatic PHs	C6–C8	Ingestion	7.65E−06	1.25E−05	2.08E−05
			Dermal	5.40E−04	8.80E−04	1.47E−03
		C9–C18	Ingestion	5.36E−05	2.37E−03	4.33E−04
			Dermal	3.78E−03	1.67E−01	3.06E−02
		C19–C32	Ingestion	3.84E−05	2.71E−03	3.87E−04
			Dermal	2.71E−03	1.91E−01	2.73E−02
		C33–C40	Ingestion	1.02E−02	1.66E−03	4.05E−04
			Dermal	7.23E−01	1.17E−01	2.86E−02
	Aromatic PHs	C6–C16	Ingestion	5.58E−05	2.11E−03	4.24E−04
			Dermal	3.94E−03	1.49E−01	2.99E−02
		C17–C32	Ingestion	4.39E−05	1.45E−03	3.60E−04
			Dermal	3.09E−03	1.02E−01	2.54E−02
		C33–C40	Ingestion	1.02E−02	1.66E−02	4.05E−04
			Dermal	7.23E−01	1.17E−01	2.86E−02
Ogale, Eleme LGA	Aliphatic PHs	C6–C8	Ingestion	1.23E−04	6.24E−06	3.85E−06
			Dermal	8.65E−03	4.41E−04	2.72E−04
		C9–C18	Ingestion	5.60E−04	1.07E−03	2.08E−04
			Dermal	3.95E−02	7.56E−01	1.47E−02
		C19–C32	Ingestion	3.88E−04	3.78E−03	2.07E−03
			Dermal	2.74E−02	2.67E−01	1.46E−01
		C33–C40	Ingestion	5.21E−04	5.14E−03	1.67E−03
			Dermal	3.68E−02	3.63E−01	1.18E−01
	Aromatic PHs	C6–C16	Ingestion	5.69E−04	3.06E−04	7.65E−05
			Dermal	4.01E−02	2.16E−02	5.39E−03
		C17–C32	Ingestion	5.02E−04	4.55E−03	2.20E−03
			Dermal	3.54E−02	3.21E−01	1.55E−01
		C33–C40	Ingestion	5.21E−04	5.14E−03	1.67E−03
			Dermal	3.68E−02	3.62E−01	1.18E−01

Table 11 shows the hazard quotient (HQ) of aliphatic and aromatic petroleum hydrocarbons (PHs) calculated using the reference dose in Table 2 for adults across three rain sampling regiments. Aliphatic PHs evaluated for R/R and O/E: HQ (ingested) varies from 7.02E−02E−05 to 1.30; HQ (dermal) varies from 4.12E−06 to 1.20E−01. Aromatic PHs evaluated for R/R and O/E: HQ (ingested) varies from 5.56E−03 to 2.89; HQ (dermal) varies from 1.49E−03 to 1.82. Critical values of aliphatic hazard quotient at R/R were C_9_–C_18_ (1.30E−01 and 2.38E−01) mid and late rain via ingestion but dermal is 1,27E−01. For aromatic PHs; C_6_–C_16_ (5.82E−01) via ingestion of late rain. C_17_–C_32_ (1.99E−01 and 1.94E−01) for ingestion and dermal contact of early rain but C_33_–C_40_ (dermal) is 1.82E + 00 and 2.96E−01 for early and late rain exposure. At Ogale study area, aliphatic critical values occurred at C_9_–C_18_ via ingestion (3.07E−01, 5.88E−01 and 1.14E−01) for early, mid and late rain. For aromatic PHs of C_6_–C_16_ through ingestion (7.80E−01, 4.20E−01 and 1.05E−01) for early, mid and late rain but for C_17_–C_32_ (6.24E−01 and 3.02E−01) and (6.08E−01 and 2.94E−01) via ingestion and dermal exposure to mid and late rain, C_33_–C_40_ (9.14E−01 and 2.99E−01) via dermal contact with mid and late rain.

**Table 11:** Hazard Quotient for adult non-risk assessment.

Location	TPHs group	TPHs derivatives	Exposure pathway	Time of early rain	Exposure mid rain	Late Rain
Rumuodomaya/ Rumuodome, Obio-Akpor LGA	Aliphatic PHs	C6–C8	Ingestion	1.05E−03	171E−03	2.85E−03
			Dermal	8.17E−06	1.33E−05	2.22E−05
		C9–C18	Ingestion	2.94E−02	1.30E−01	2.38E−01
			Dermal	2.86E−03	1.27E−01	2.31E−02
		C19–C32	Ingestion	7.02E−05	4.96E−03	7.08E−04
			Dermal	1.03E−04	7.24E−03	1.03E−03
		C33–C40	Ingestion	1.87E−03	3.04E−04	7.40E−05
			Dermal	2.73E−03	4.43E−04	1.08E−04
	Aromatic PHs	C6–C16	Ingestion	7.65E−02	2.89E + 00	5.82E−01
			Dermal	1.49E−03	5.63E−02	1.13E−02
		C17–C32	Ingestion	6.01E−03	1.99E−01	4.93E−02
			Dermal	5.85E−03	1.94E−01	4.80E−02
		C33–C40	Ingestion	1.40E−01	2.28E−02	5.55E−03
			Dermal	1.82E + 00	2.96E−01	7.21E−02
Ogale, Eleme LGA	Aliphatic PHs	C6–C8	Ingestion	1.68E−02	8.56E−04	528E−04
			Dermal	1.31E−04	6.67E−06	4.12E−06
		C9–C18	Ingestion	3.07E−01	5.88E−01	1.14E−01
			Dermal	2.99E−02	5.72E−02	1.11E−02
		C19–C32	Ingestion	7.09E−04	6.92E−03	3.78E−03
			Dermal	1.04E−03	1.01E−02	5.52E−03
		C33–C40	Ingestion	9.53E−05	9.39E−04	3.06E−04
			Dermal	1.39E−04	1.37E−03	4.47E−04
	Aromatic PHs	C6–C16	Ingestion	7.80E−01	4.20E−01	1.05E−01
			Dermal	1.52E−02	8.18E−03	2.04E−03
		C17–C32	Ingestion	6.88E−02	6.24E−01	3.02E−01
			Dermal	6.70E−02	6.08E−01	2.94E−01
		C33–C40	Ingestion	7.15E−03	7.04E−02	2.29E−02
			Dermal	9.28E−02	9.14E−01	2.99E−01

Table 12: Hazard quotient (HQ) of aliphatic and aromatic petroleum hydrocarbons (PHs) calculated using reference dose in Table 2 for children across three rain-sampling regiments. Aliphatic PHs evaluated for R/R and O/E: HQ (ingested) ranged from 1.28E−05 to 2.37E−01; HQ (dermal) ranged from 5.44E−05 to 1.67. Aromatic PHs were HQ (ingested) varies from 1.01E−3 to 5.28E−01; HQ (dermal) varies from 1.97 to 24.09. Hazard quotient critical values of aliphatic for C_9_–C_18_ (1.67E + 00 and 3.06E−01) via dermal route for mid and late rain. When considering aromatic C_6_–C_16_ (5.28E−01, 1.06E−01) via ingestion for mid and late rain at R/R area. At Ogale area, aliphatic PHs C_9_–C_18_ (7.56E−01 and 1.47E−01) via dermal for mid and late rain. Aromatic PHs critical valued are C_6_–C_16_ (1.42E−01 and 2.01E−01) for ingestion and dermal exposure to early rain. C_17_–C_32_ (8.49E−01, 8.03E + 00, 3.89E + 00) for dermal exposure to early, mid and late rain, the same was observed for C_33_–C_40_ via dermal for the sampling regimes.

**Table 12:** Hazard quotient for children non-risk assessment.

Location	TPHs group	TPHs derivatives	Exposure medium	Early rain	Mid rain	Late rain
Rumuodomaya/Rumuodome, Obio-Akpor LGA	Aliphatic PHs	C6–C8	Ingestion	1.91E−04	3.12E−04	5.20E−04
			Dermal	1.08E−04	1.76E−04	2.93E−04
		C9–C18	Ingestion	5.36E−03	2.37E−01	4.33E−02
			Dermal	3.78E−02	1.67E + 00	3.06E−01
		C19–C32	Ingestion	1.28E−05	9.05E−04	1.29E−04
			Dermal	1.35E−03	9.57E−02	1.37E−02
		C33–C40	Ingestion	3.41E−04	5.54E−05	1.35E−05
			Dermal	3.61E−02	5.86E−03	1.43E−03
	Aromatic PHs	C6–C16	Ingestion	1.40E−02	5.28E−01	1.06E−01
			Dermal	1.97E−02	7.44E−01	1.50E−01
		C17–C32	Ingestion	1.10E−03	3.63E−02	8.99E−03
			Dermal	7.73E−02	2.56E + 00	6.34E−01
		C33–C40	Ingestion	2.56E−02	4.15E−03	1.01E−03
			Dermal	2.41E + 01	3.91E + 00	9.52E−01
Ogale, Eleme LGA	Aliphatic PHs	C6–C8	Ingestion	3.07E−03	1.56E−04	9.63E−05
			Dermal	1.73E−03	8.81E−05	5.44E−05
		C9–C18	Ingestion	5.60E−02	1.07E−01	2.08E−02
			Dermal	3.95E−01	7.56E−01	1.47E−01
		C19–C32	Ingestion	1.29E−04	1.26E−02	6.89E−04
			Dermal	1.37E−02	1.33E−01	7.29E−02
		C33–C40	Ingestion	1.74E−05	1.71E−04	5.58E−05
			Dermal	1.84E−03	1.81E−02	5.90E−03
	Aromatic PHs	C6–C16	Ingestion	1.42E−01	7.66E−02	1.91E−02
			Dermal	2.01E−01	1.08E−01	2.70E−02
		C17–C32	Ingestion	1.25E−02	1.14E−01	5.51E−02
			Dermal	8.49E−01	8.03E + 00	3.89E + 00
		C33–C40	Ingestion	1.30E−03	1.28E−02	4.18E−03
			Dermal	1.23E + 00	1.21E + 01	3.93E + 00

**Figure 1:**
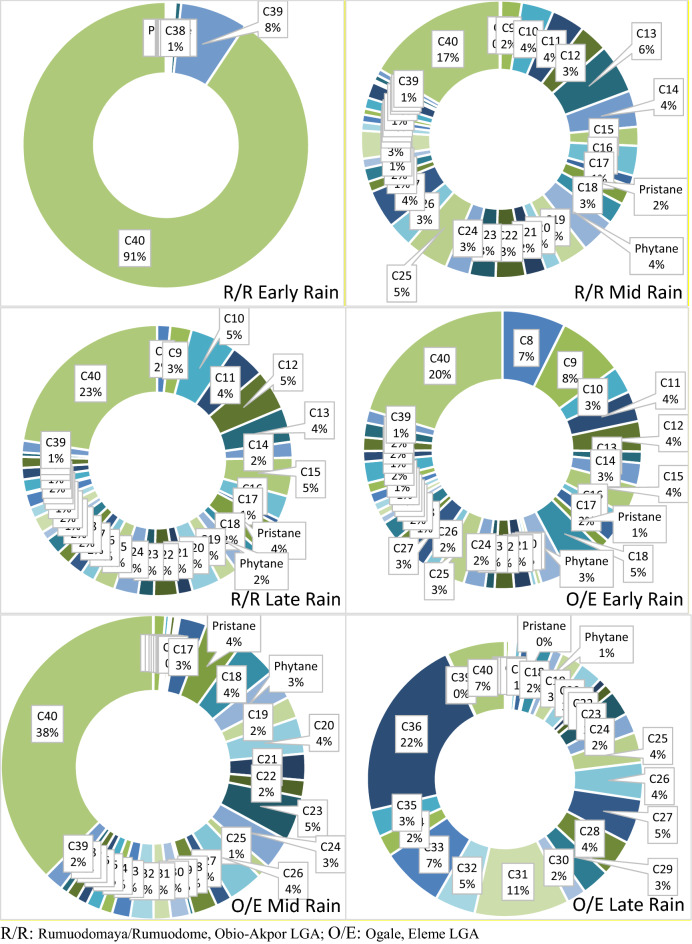
Percentage composition of TPHs across sampling period. *R/R* Rumuodomaya/Rumuodome, Obio-Akpor LGA; *O/E* Ogale, Eleme LGA.

Figure 1 shows the percentage of TPHs in ambient rainwater samples in R/R and O/E sampling regiments. For R/R early rain, C40 had over 90.66% cumulative TPHs concentration in tandem with C39 having 7.61%, while C8–C38 shared 1.73% accordingly. For R/R mid rain, C40 had the highest TPHs concentration of 16.58%, as C13 and C25 were above 5%, while C14, Phytane, C27 were within 4.12–4.47%, as other TPHs aggregates ranged from 0.17–3.90%. R/R late rain displayed that C40 was 22.662%, as compared to other TPHs (C8–C39) aggregates ranging from 0.20–5.48%. O/E produced the highest percentage TPHs of C40 (20.38%), C40 (37.42), and C36 (21.66) for the three rain sampling regiments (early rain, mid rain, and late rain), while the least percentage was present in C19 (0.55), C16 (0.05) and C37 (0.02) accordingly.

**Figure 2:**
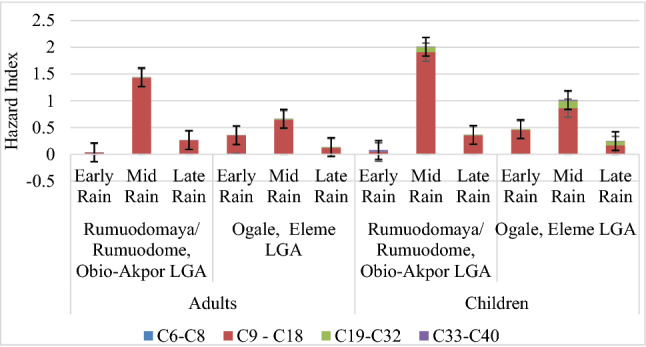
Hazard index of adults and children for aliphatic petroleum hydrocarbons.

Figure 2 displays results of hazard index (HI) evaluated from HI (ingested and dermal) for aliphatic petroleum hydrocarbons (PHs) in adults and children. The cumulative sum of all HI (C6–C8, C9–C18, C19–C32 and C33–C40) assessed across the three rain-sampling regiments are Adults: R/R (0.038, 1.441, and 0.266), O/E (0.3561, 0.665, and 0.136), Children: R/R (0.081, 2.012 and 0.365), O/E (0.472, 1.016 and 0.247).

**Figure 3:**
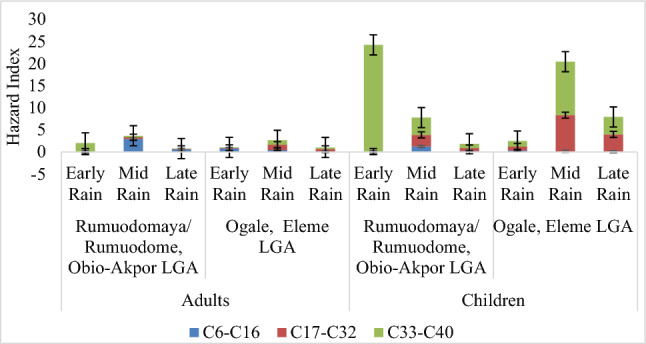
Hazard index of adults and children for aromatic petroleum hydrocarbons.

Figure 3 shows the results of hazard index (HI) calculated from HI (ingested and dermal) for aromatic petroleum hydrocarbons (PHs) in adults and children. The cumulative sum of all HI (C6–C16, C17–C32, C33–C40) across early rain, mid rain, and late rain are Adults: R/R (2.054, 3.661 and 0.768), O/E (1.031, 2.646 and 1.024), Children: R/R (24.226, 7.779 and 1.852), O/E (2.468, 20.424, 7.925).

## Discussion

### Level of total petroleum hydrocarbons (TPHs) in ambient atmospheric rainwater

Rivers State of Nigeria over the years has been embroiled in numerous cases of crude oil drillings, spills and illicit pipeline bunkering, crude oil, its fractional derivatives and gas pipeline vandalization, massive gas flares, hydrocarbon installation fire outbreaks, refinery operations, these activities are known to release toxic gaseous hydrocarbons into the atmosphere^35^, related emissions from associated-petroleum industries include automobiles, coastal marine transportation, homes, and manufacturing industries utilizing diesel generators, burn-pits also account for total petroleum hydrocarbon (TPH) concentration in the atmosphere^36–38^. All the aforementioned releases in the atmosphere undergo chemical interactions, several induced or natural chemical processes such as volatilization, photo-oxidation, and biodegradation, which combine with air moisture before atmospheric rainfall deposition impacting soil and surface water quality^39,40^. The surface water of the present study area is known to be laden with conjugated and straight-chain hydrocarbon^17,41^, most of which come from atmospheric deposition via air and rainfall.

Results showed that ambient rainwater at Rumuodomaya/Rumuodome (R/R) and Ogale (O/E) had elevated levels of total petroleum hydrocarbons in their combined forms (aliphatic and aromatic petroleum hydrocarbons) from early rain to late rain (Table 3). The TPH concentration at Rumuodomaya/Rumuodome decreased from early rain to late rain, while Ogale had a high concentration at mid rain, least for late rain that reveals the rainwater were relatively contaminated and unsafe for human consumption from crude oil and gas processing releases into the atmosphere and subsequently as rainwater. Using USEPA^42^, and TPHCWG^19^, petroleum hydrocarbons categories, there was the presence of both light and heavy carbon chains (C8–C40). C19–C40 indicates the presence of fuel oils; other carbon groups are due to anthropogenic activities. The highest TPH concentrations was predominant for C40 across all locations and sampling regimens and shows that higher molecular weight hydrocarbons were present. Specific range from Table 3 as carbon chain length shows TPHs value of 0.0046 of C21–TPHs (56.6551) of C40 (early rain); 0.0683 of C8–TPHs of 6.5533 of C40 (mid rain) and 0.0142 of C34–1.6351 (late rain) at Rumuodomaya/ Rumuodome (R/R) while at Ogale (O/E), it ranged from 0.0500 of C19–1.8601 of C40 (early rain), 0.0342 of C8–22.237 (mid rain) and 0.0050 of C37–1.511 (late rain). A look at Table 3 shows that variation exist between sampling period and study sites. The likely reason may be because of transboundary movement of petroleum hydrocarbons particulates that were in the atmosphere and closeness to source and intensity of emission. Also, the study area lies on long coastal area of the Atlantic Ocean, at early rain, there may be high concentration of gaseous pollutants, as intensity and volume of rain increases, dilution effect can occur. Again, since it is a coastal region of Atlantic Ocean, the humidity may be above average throughout the year and may cause local variation. Similar studies by Ali et al.^10^ showed the presence of TPHs in River water in significant levels in three-sampling stations as they got lower concentration in October 2018, while January 2019 had a higher concentration in Al-Gharraf, Iraq, which was attributed to emission from sewage releases into rivers and municipal wastes from cities and farmland but the levels obtained were lower in comparison to the TPHs values presented here. According to Kennedy et al.^3^, rainfall reveals the overall and regional atmospheric quality inclusive of all forms of emissions to the atmosphere, as similar assessment by Griffiths and Timperley^43^ noted the presence of visible oil sheens on vehicle screens with particulate matter. Rainfall intensity and duration known to vary in pattern, may have influence as continuous rain events takes place, the concentration reduced continuously from high to low and vice versa due to the seasonal pattern (wet and dry) peculiar with Nigeria, in addition, the influence of human activities such as helicopter, aeroplane fuel discharge on air transit, anaerobic microbial events in waste dumps and marine organisms, flue gas burning, marine vessels and transports, pyrolysis and petrogenic events that are transboundary from offshore (Atlantic ocean) to onshore (land) from wind movement (South to Northwest and Eastward) may influence petroleum hydrocarbons concentration.

### Chemometric assessment

#### Contamination factor and pollution load index

Contamination factor (CF) and pollution load index (PLI) were conducted on aliphatic and aromatic hydrocarbons within THGCWG^19^ standards as shown in Table 4, aliphatic hydrocarbons via both locations, C5–C8 were < 1, implying low contamination, while other carbon aggregates (C9–C16, C17–C35 and C36–C40) indicates high contamination except for early and late rain of C17–C35 and C36–C40 at Rumuodomaya/Rumuodome (R/R), also early and late rain of C36–C40 at Ogale (O/E) respectively. Considering aromatic hydrocarbons, C5–C8 at the R/R location were below one (1) while that of O/E early rain was considered contaminated, other carbon aggregates were highly present. Using PLI assignment, aliphatic hydrocarbons at R/R early rain were within safe background level except for mid and late rain that were above one (1), indicating high pollution of atmospheric rainwater, at Ogale (O/E), rainwater was highly polluted for all three rain sampling periods but aromatic hydrocarbons at both R/R and O/E were high and polluted as they were above one (1)^44^. The contamination of water by TPH is associated with increase in particulate matter of fine particle size, high temperature, reduced dissolved oxygen, low salinity, and anaerobic reactions^45^. In addition, TPHs at higher carbon ranges are known to form oily films that prevent limited sunlight penetration thus influencing poor water quality leading to taste and odour issues over time.

#### Principal component analysis

Total petroleum hydrocarbons (TPHs) were subjected to principal component analysis using rotated varimax as seen in Table 5. Principal component analysis is a statistical tool used to aggregate data set to a linear regression (y = mx + C) between 0.00–0.99, which is computed using Liu et al.^23^ categories (strong, medium and weak regression). Rotated varimax was conducted as Rumuodomaya/ Rumuodome (R/R) had two factors with 29.50% cumulative variance, while Ogale (O/E) had 77.89% cumulative variance. There were positive and negative TPHs components across the two factors assigned. Using Liu et al.^23^ at R/R, Factor 1 had moderate and strong regression grades except for C37 and C40, which had weak regression, while Factor 2 had moderate and strong regression, at O/E Factor 1 had predominant weak and strong regression across all TPHs components assessed except C31, while Factor 2 had strong, moderate and weak regression except for C16–C27 and C37–C40 that were very weak (< 0.30). As we can infer across both locations (R/R and O/E), positive components are due to the presence of the TPH components from varying sources either petrogenic or phytogenic petroleum sources. The negative components can be due to atmospheric reactions with pyrogenic petroleum sources (combustion of carbonaceous substances), marine sea sprays, and climatic conditions^46^.

### Petroleum hydrocarbon source identification

#### Carbon preference index

Carbon preference index (CPI) shown in Table 6, indicated that values above one (1) were due to natural sources (terrestrial vascular floras) as compared to CPI below one (1), more in number that was due to anthropogenic and petroleum activities from the combustion of crude oil, gas flares, refinery and petrochemical plant, emission of organics from industries as a major economic activity within the area. The TPHs aggregates at Rumuodomaya/ Rumuodome (R/R) were highest at C8–C11 (1.034 and 1.005) early rain. C18–C25 and C26–C33 have CPI of 1.287 and 1.630 (mid-rain). C26–C33 has a CPI of 1.288 (late-rain) but at Ogale area, C26–C33 has the highest CPI value of 1.732 (early-rain), the mid-rain highest value is for C8–C11 (2.768) and C12 –C17 (5.368) while late-rain exhibited a CPI value of 1.490, 1.592, 1.193 and 1.728 (C8–C11, C12–C17, C18–C25 and C26–C39), all these were greater than CPI value of one (1) but accounted for only 40% while values less than one (1) represent 60% hence CPI were dominated by anthropogenic origin of petroleum processing ^13,47^.

#### Average carbon length (ACL)

Average carbon length (ACL) values as evaluated (Table 7) fluctuated for early rain as compared to mid rain and late rain and varied slightly for odd n-alkanes, showing there was a little anthropogenic contribution to the odd carbon aggregates as compared to early rain respectively^48^. ACL of TPHs aggregates shows that for odd n-alkanes minimum and maximum values were C9–C11 (9.446) and C35–C39 (38.980), C9–C11(10.238) and C35–C39 (36.510); C9–C11 (10.240) and C35–C39 (36.934) for early, mid and late rain respectively. Considering even n-alkanes, the same trend of C8–C10 (9.086) and C34–C40 (39.985); C8–C10 (9.912) and C34–C40 (39.985); C8–C11 (9.553) and C34–C40 (39.688) at Rumuodomaya/ Rumuodome (R/R) but for Ogale (O/E) area, the ACL of TPHs aggregates of odd n-alkanes were C9–C11 and C35–C39 (9.631 and 36.647; 9.323 and 37.210; 9.362 and 35.115) for early, mid and late rain. Looking at even n-alkanes, C8–C10 and C34–C40 (8.596 and 39.365; 9.740 and 39.660; 9.240 and 36.727) for early, mid and late rain, again for n-alkanes, similar variation as seen in odd n- alkanes were attributed to slight anthropogenic input^49^. The results of odd ACL vs CPI plots for early rain, mid rain, and late rain reveal that regression (R^2^) for Rumuodomaya/ Rumuodome (R/R) were 0.4264, 0.0067, 0.0048, while O/E were 0.0103, 0.5062, 0.3358 indicating an increase in ACL as CPI values fluctuate. The regression for even-n-alkanes ACL/CPI for R/R were 0.4882, 0.0389, 0.0266, O/E were 0.0242, 0.5976, 0.4357 signifying similar trends for odd-n-alkanes. This confirms that CPI assessments were influenced by both natural and anthropogenic sources.

#### Petroleum source diagnostics

Petroleum hydrocarbon source diagnostic ratio were assessed and depicted in Table 8. with an aim to assess possible source across different environmental matrices, floras and faunas. Average carbon length (ACL) (odd n-alkanes) has the highest value of 37.854 (early rain) and 28.248 (late rain) for Rumuodomaya/ Rumuodome (R/R) and Ogale (O/E) sampling sites. ACL (even n-alkanes) has 39.854 and 33.565 for early and mid-rain of both study areas. The two location’s long-chain hydrocarbons/short-chain hydrocarbon ratio (LHC)/SHC) of odd n-alkanes and LHC/SHC of even n-alkanes, the highest values were as follows: 22.268, 220.33 (early rain), and 4.019, 4.881 (late rain). C31/C19 showed that all three rainfall events evaluated were above 0.4, thus shows the impact of land sources as an array of emissions from crude oil processing, industrial flue gases, power plants, automobile, heavy-duty vehicles, and flare gases^50^. In addition, elevated temperature in the soil releases volatile organic compounds while decreases in temperature usually at night activate microbial decomposition of organic matter leads to the production of petroleum hydrocarbons, influences the TPH concentration in the atmosphere. Low molecular/high molecular (L/H) weight n-alkanes evaluated showed that the L/H ratio was below one (1), suggesting impact from phytogenic and pyrogenic sources. Long-chain hydrocarbons/short-chain hydrocarbons (LHC/SHC) assessed for odd n-alkanes reveals that at R/R early rain and O/E late rain were above one (1), indicating terrestrial floral sources, while below 1 is due to phytoplankton sources. LHC/SHC (even n-alkanes) were all above one (1), thus confirming all were from (anthropogenic) petroleum sources (Table 8). Cumulative CPI assessed were below one (1) from a pyrogenic source. Cumulative average carbon length (ACL) (odd and even n-alkanes) showed fluctuating values confirming impact from anthropogenic sources. The plot of cumulative odd ACL against cumulative CPI via the influence of the three rain regimens showed that regression, R^2^ were 0.9454 and 0.0607 at R/R and O/E, while even ACL versus CPI plot gave regression, R^2^ = 0.9594 and 0.291 respectively, hence depicting that petroleum hydrocarbon in atmospheric rainwater of the study area may indicate non-marine and anthropogenic sources. The ratio of pristane/phytane exceeded one (1) for R/R early rain and late rain in relation to O/E mid rain indicating biogenic (aerobic) sources, as anaerobic indices are possible for values less than one (1) for R/R mid rain and O/E early and late rains^51^. In addition, different assessment has shown that pristane/phytane less than 0.8 implies saline to the hypersaline condition due to extreme evaporation and carbonaceous deposition, whereas pristane/phytane above 3 is due to oxygenated to non-oxygenated degradation of planktons from anaerobic organisms^52^. Pristane and phytane are bi-products of chlorophyll in the aquatic environment, pristane are produced from the breakdown of zooplankton and phytoplankton (algae) in an oxygenated aquatic environment to form lipids, whereas phytanes are from anaerobic degradation of aquatic planktons by microbial organisms (cyanobacteria)^53^. Therefore, we can infer that petroleum hydrocarbon in atmospheric rainwater of the study area were from diverse sources (natural and human activities) that have the potential to impact on rainwater quality in relation to environmental releases such as microbial releases due to anaerobic condition in aquatic or waste dump areas, petroleum spills, flue gases, natural gas release into the atmosphere from earth sources and pyrolytic activities.

### Risk assessment

A serious problem of the study area is non availability of potable water especially in the rural communities, even as the surface and underground water of the area are contaminated^16,17,41^, hence rain water becomes aa available alternative. Average daily intake (ADI) of aliphatic and aromatic petroleum hydrocarbons aggregates was assessed for ambient atmospheric rainwater from Rumuodomaya/Rumuodome (R/R) and Ogale (O/E). Two exposure pathways were calculated using ingestion (oral) and dermal (skin contact) to assess the average daily intake (ADI) of rainwater for adults and children as depicted in Tables 9 and 10, but from Table 9, aliphatic PHs of C33–C40 were exposed to adult through dermal and ingestion by 5.47 × 10^–02^ and 5.62 × 10^–02^ via consumption of early rain at Rumuodomaya/Rumuodome, the same for aromatic PHs of C33–C40 (5.47 × 10^–2^ and 5.62 × 10^–2^). At Ogale, the same adults are more at risk of aliphatic PHs of C19–C32 and C33–C40 (2.07 × 10^–2^, 2.02 × 10^–2^; 2.82 × 10^–2^, and 2.74 × 10^–2^) through ingestion or dermal contact with mid-rain while they are highly exposed to C17–C32 and C33–C40 by 2.50 × 10^–2^; 2.04 × 10^–2^; 2.82 × 10^–2^ and 2.74 × 10^–2^ on contact with mid-rain through ingestion and dermal pathway and to C17–C32 (the same exposure route) risk value of 1.21 × 10^–2^ and 1.18 × 10^–2^ (late rain). Non-carcinogenic risk assessment for adults and children evaluated having obtained hazard quotient (HQ) for exposed pathways as shown in Tables 11 and 12. Preliminary assessment of ADI and subsequent evaluation of HQ shows that children had elevated values compared to adults. Hazard index (HI) as shown in Fig. 1 for aliphatic petroleum hydrocarbons reveals that at R/R, mid rain for adults and children were above one (1), thus there is an inherent health risk. At O/E, mid rain for children was above one (1), as compared to adults. The hazard index for aromatic petroleum hydrocarbons shown in Fig. 2 shows that all locations including the rain period were above one (1). Risk-based mapping using carbon ranges were proposed by MADEP^54,55^ and TPHCWG^56^ to assess the health-based risk over a period for all age grade, sex orientation, diet, family traits, lifestyle and current state of health to derive salient points for regulatory consideration and action plan models. The analysed HI values reveals that ingestion of TPHs contaminated rainwater by both adult and children leads to bioaccumulation resulting in disruption of biochemical and physiological activities in the human body causing negative health outcomes usually after a short period^11^.

In the atmosphere, where free chloride ions exist in the presence of ultra-violet radiation and high-temperature conditions permit (Table 13), combines with aliphatic petroleum hydrocarbons (aerosols) to form toxic polychlorinated–n –alkanes (PCAs), and polychlorinated biphenyls (PCBs). These subsequently dissolved in rainwater impacting water quality as when ingested over a period of time can cause detrimental health issues such as liver and kidney dysfunctions, dermatitis, dizziness, and severe headaches^17^. In addition, PAHs photochemical reactions form diones, nitro-PAHs, dinitro-PAHs, and other PAHs components known to cause carcinogenic and mutagenic effects and resulting in human health crises such as bone suppression and decreased blood cell production with reproductive complications^57^.

**Table 13:** shows values of temperature and chloride ion in ambient rain water samples within the area.

Type of parameter	OGALE, ELEME LGA			RUMUDOMAYA/RUMUODOME, OBIO-AKPOR LGA		
	Early rain	Mid rain	Late rain	Early rain	Mid rain	Late rain
Temperature (ºC)	22.57 ± 0.26	24.00 ± 0.90	25.50 ± 0.10	22.87 ± 1.04	24.25 ± 0.35	25.55 ± 0.05
Cl^–^ (mg/L)	4.77 ± 4.55	1.17 ± 0.71	0.71 ± 0.21	5.33 ± 4.60	2.01 ± 0.11	0.58 ± 0.38

TPHs ingestion via atmospheric rainwater has been associated with headaches, fatigue, nausea, diarrhoea, and irritation of gastrointestinal tracts over a long period^58^. Renal disorder, digestive disruption, and interstitial oedema are also negative health outcomes associated with consumption of contaminated rainwater^59^. Dermal contact with contaminated rainwater sources can cause skin inflammation and rashes, dermatitis, blisters, eye irritation, and degreasing burns where been reported by persons during bathing periods in amazon, Ecuador^60^. In children, the presence of dandruff and psoriasis has been attributed to TPHs^61^. Petroleum hydrocarbon represents all aliphatic and aromatic petroleum formulations. Aliphatic petroleum hydrocarbon is known to cause neurological disorders, cancer of the mouth, stomach, and uterine disorders^60^. Aromatic petroleum hydrocarbons in the form of benzene, toluene, polycyclic aromatic hydrocarbons cause haematological, immunological, neurological, gastrointestinal disorders inclusive of possible death associated with these contaminants. This study forms the basis of call to policy makers in Nigeria on the need to provide potable water to its citizens, especially in the crude oil and gas rich region (Niger Delta area), lack of potable water has forced the inhabitant to depend on surface and rainwater for drinking and domestic needs with its attendant health risks. Unachukwu and co-worker^62^ reported that non-communicable diseases (NCDs) such as cardiovascular disease, diabetes mellitus, cancer, renal diseases, liver failure and so on, which may be associated with oil pollution are now highly diagnosed and reported in hospitals within the study area and may be more pronounced amongst poor rural dwellers. Having assessed these possible health effects, children are most at risk as reports by Steven^60^, give worrisome reports that need adequate attention to mitigates these issues in the long run.

## Conclusion

The present study assessed total petroleum hydrocarbons (TPHs) in rainwater sampled via three regimens (early rain, mid rain, and late rain) at Rumuodomaya/ Rumuodome and Ogale in Rivers State, Nigeria. The TPH concentration at Rumuodomaya/ Rumuodome decreased from early rain to late rain, while Ogale had a high concentration at mid rain, least for late rain that reveals the rainwater were relatively contaminated and unsafe for human consumption due to crude oil and gas processing releases into the atmosphere and subsequently as rainwater. Chemometric assessment using total petroleum hydrocarbon criteria working group standards showed that both aliphatic and aromatic petroleum were relatively high. TPHs source identification showed that carbon preference index and average carbon length gave correlation, which implies that contaminations were due to anthropogenic sources, probably hydrocarbon been the major economic activity in the area. A risk assessment conducted showed that hazard index was above one (1) for aromatic petroleum hydrocarbons compared to aliphatic petroleum hydrocarbon that had varying levels. The pollution levels show that children were more at risk from continuous oral and dermal exposure to TPHs in rainwater. Government agencies with assistance from oil and gas stakeholders can set up a monitoring stations for air, soil, and water contamination sources to assist in the development of a cost-effective approach and remediation action plan to avert possible health issues in the future.

## Data Availability

We have no special data information to declare, every data pertaining to the work is as presented in the results.
